# Utilization of potentially inappropriate medication and risk of adverse drug events among older adults with chronic renal insufficiency: a population-wide cohort study

**DOI:** 10.1186/s12877-021-02057-5

**Published:** 2021-02-10

**Authors:** Safoura Sheikh Rezaei, Hana Šinkovec, Alexander Schöberl, Christoph Rinner, Georg Heinze, Michael Wolzt, Walter Gall

**Affiliations:** 1 Department of Clinical Pharmacology, Medical University of Vienna, Spitalgasse 23, 1090 Vienna, Austria; 2 Section for Clinical Biometrics, Center for Medical Statistics, Informatics and Intelligent Systems, Medical University of Vienna, Spitalgasse 23, 1090 Vienna, Austria; 3 Section for Medical Information Management, Center for Medical Statistics, Informatics and Intelligent Systems, Medical University of Vienna, Spitalgasse 23, 1090 Vienna, Austria

**Keywords:** Medications, Chronic kidney disease, Adverse drug events, Pharmaco-epidemiology, Older adults

## Abstract

**Background:**

The use of potentially inappropriate medication (PIM) in population of older adults may result in adverse drug events (ADE) already after short term exposure, especially when it is prescribed to patients with chronic kidney disease (CKD). In order to limit ADE in the treatment of older adults PIM lists have been constructed as a source of information for healthcare professionals. The aim of this study was to estimate the utilization of PIM and incidence of ADE in older adults (≥70 years) with CKD.

**Methods:**

We conducted a retrospective population-wide cohort study including patients from Lower Austria who were 70 years or older and diagnosed with CKD in the period from 2008 to 2011. Utilization of PIM was estimated from prescriptions filled by target population. We estimated risks of hospitalization due to ADE within 30 days after incident PIM prescription and compared them to a PIM-free control group by using marginal structural models (MSM).

**Results:**

We identified 11,547 patients (women: 50.6%, median age in 2008: 78 years) who fulfilled the inclusion criteria. In total 24.7 and 8.1% of all prescriptions from that period contained a medication with a substance listed in the EU (7)-PIM and AT-PIM list, respectively. Proton pump inhibitors and *Ginkgo biloba* were the most often prescribed PIMs in this population. 94.6 and 79.3% patients filled at least one EU(7)-PIM and AT-PIM prescription, respectively. Despite the relatively high utilization of PIM there was only a low incidence of clinically relevant ADE. No event type exceeded the threshold level of 1% in the analysis of risks of ADE after filling a prescription for PIM. Nevertheless, MSM analysis showed an increased risk for 11 drugs and reduced risk for 4 drugs.

**Conclusions:**

PIM prescription was common among older adults with CKD, however, only a small number of these drugs eventually led to hospitalization due to ADE within 30 days after incident PIM was filled. In the absence of a clinically important PIM-related increase in risk, an assessment of potential ADE severity to a PIM list by using a warning score system seems prudent.

**Supplementary Information:**

The online version contains supplementary material available at 10.1186/s12877-021-02057-5.

## Background

The prevalence of chronic kidney disease (CKD) is increasing, which affects aging populations especially [1]. CKD is associated with an increased risk of adverse drug events (ADE), particularly when older adults with multiple comorbidities are exposed to polypharmacy [2, 3]. Older adults are predisposed to develop acute kidney injury caused by dehydration or pre-existing kidney disease. Renal failure and aging process have an impact on pharmacokinetic and pharmacodynamic profiles of drugs and can result in an increased incidence of ADE [4]. Dose adjustments are therefore necessary for some drugs and their metabolites, which are excreted by the kidney in patients with CKD, to prevent potential ADE. In some cases, the treatment, e.g. metformin, has to be discontinued, if serious side effects occur [5].

The use of potentially inappropriate medication (PIM) can lead to adverse drug events (ADE) and is the cause of major health concern in older adults [6, 7]. In Austria, a consensus-based list of potentially inappropriate drugs (AT-PIM list) serves as a source of information for healthcare professionals to limit ADE in the treatment of geriatric patients [8]. A widely accepted consensus list was developed in the US in 1991, which is known as “Beers-list” [9]. In 2015, in cooperation with experts from several countries, the EU(7)-PIM list was established, which contains 330 different substances [9, 10]. The AT-PIM list [11] that contains 75 substances is based on the German PRISCUS list of PIM [12] and is customized to prescription practice in Austria.

The utilization of PIM assessed by claims data of certain health insurances varies across countries [2, 7, 13–15]. Alarming is that in 2012 over 72% of older adults in nursing homes in Austria received at least one PIM during observation period of 30 days [16]. This number is similar to a previous investigation in France [17]. Antidepressants, antipsychotics and nonsteroidal anti-inflammatory drugs (NSAIDs) have been reported to be the most prescribed substances in patients over 80 years in Austria [16]. Only few studies have investigated the prescription patterns of inappropriate medications in elderly patients with CKD [1, 18], the population of patients that is especially vulnerable and likely under high risk of ADE if PIM is prescribed. No study focused on PIM utilization neither estimated the risks of ADE due to PIM in older adults with CKD in Austria. The estimation of causal relationships in observational data has seen a lot of new developments in recent research, going far beyond simple descriptions and comparisons [19]. For example, marginal structural models (MSM) can provide estimates of causal effects of treatments that vary over time, but their application remains challenging with registry data [20]. In this study we have developed and applied the necessary methodology for estimating the risks of ADE after filling a PIM prescription using a large registry of prescriptions and hospitalizations.

The aims of this study were therefore defined as follows: 1) to estimate the utilization of PIM contained in EU(7)-PIM as well as AT-PIM lists in the population of older adults with CKD in Lower Austria, which is the country’s largest and second most populous province; 2) to describe the risk of ADE within 30 days after a prescription of PIM was filled; 3) to apply MSM in order to attribute increased risk of ADE to filling a prescription of PIM relative to a PIM-free control group; 4) to investigate whether any other substances that are not included in the PIM lists increased the risk of ADE.

The paper is structured as follows: Methods section describes the methodology including study design and setting, the inclusion criteria defining the study population, outcomes and exposures of interest and statistical methods used for the analysis; subsequently, the results reporting the utilization of PIM and risk of ADE after a prescription of PIM or other medications are summarized; finally, our main findings together with strengths and limitations of the study are discussed.

## Methods

### Study design, setting, sample size

The health insurance is mandatory in Austria and healthcare is provided for all residents who are assigned membership in one of several health insurance funds depending on their current or former employment or province of residence. Data on medical services covered by the health insurance funds are collected in routine databases run by the Main Association of Austrian Social Insurance Institutions. For scientific purposes, data from the provincial health insurance fund of Lower Austria from 2008 to 2011 have been prepared in the database GAP-DRG2 [21]. Containing data from the second largest provincial sickness fund of Austria, GAP-DRG2 covers approximately 14% of the population of Austria. About 71% of the Lower Austrian population is covered by this sickness fund. Only federal employees, farmers, independent entrepreneurs, and railway and mining employees are not covered.

The scientific database includes demographic data of the insured patients, their filled drug prescriptions, and, if applicable, any hospital discharge records containing date of discharge, length of stay, and primary and associated diagnoses coded using the 10th revision of the International Statistical Classification of Diseases and Related Health Problems (ICD10) system. Each medication is described by a unique Austrian pharmaceutical registration number linked to the Anatomical Therapeutic Chemical (ATC) Classification System, and for each prescription the ATC code, the volume and the specialty of prescriber (general practitioner or specialist) are recorded. We made use of these data to describe utilization of PIM in Austria and the risk of hospitalization due to ADE after a prescription of incident PIM in a retrospective cohort study. To correctly specify the temporal order of events, the study cohort was defined separately for each research aim. Analyses were carried out independently for each PIM from the EU(7)-PIM list and AT-PIM list, respectively. The study cohorts were selected by queries from the database applying the inclusion criteria detailed below. Reliability of the retrieved data was assessed by comparison of marginal frequencies with expectations from clinical expertise.

### Participants

Patients aged 70 years or older who were discharged from a hospital with the principal or associate diagnosis of CKD were eligible for this study. CKD was defined based on ICD10 codes acute, chronic, and unspecified renal failure (N17, N18, and N19), including sub-codes. The following ATC codes and sub-codes of the substances, which are commonly prescribed to patients with CKD, were also used as an indication of CKD: drugs for treatment of hyperkalemia and hyperphosphatemia (V03AE), other antianemic preparations (B03XA), and A11C vitamin D and analogues (including A11CC01, A11CC02, A11CC03, A11CC04, A11CC06, A11CC20, A11CC55). In addition, to identify patients with CKD we followed the procedure developed based on diagnoses from hospitals and sick leaves to predict the ICD code from the ATC code [22]. Patients with a predicted probability of at least 0.9 of N17, N18 or N19 were also included in the study. In addition, an observational period of at least 90 days prior to PIM prescription needed to be available for each patient.

### Issue of interest

Those (PIM) prescriptions were considered that were filled by a patient after a washout phase of at least 90 days during which the patient must not have filled a prescription for the same PIM. By this criterion we limited the investigation to ADE that occurred due to incident drug therapy but were more certainly related to one PIM as most of the studied patients were multimorbid and were filling many prescriptions simultaneously. The utilization of PIM contained in EU(7)-PIM as well as AT-PIM lists among older adults was described from several different aspects: as the proportion of filled prescriptions that contained a medication listed in the PIM lists, the proportion of patients that filled a prescription of a medication listed in PIM lists as well as the proportion of individual PIM prescribed out of all PIM contained in the lists. Frequencies of prescriptions of PIM were compared by specialty of prescriber (general practitioner or specialist).

### Endpoint

ADE were defined based on 448 ICD10 codes (a list is contained in the supplement) and were not PIM specific. The ADE-relevant diagnoses were taken from Stausberg et al. who defined it for Germany [23], and adapted for studies exploring ADE in Austria [24, 25]. In addition, we considered only those ADE as relevant that occurred within 30 days after filling a prescription of any PIM.

### Comparison

The MSM methodology demands that the treated and control groups are defined dynamically: at each specific time point and for each PIM the treated patients were those who filled a prescription for that PIM after fulfilling the inclusion criteria and the control group were all other patients who had by the time not (yet) filled a prescription for that PIM. Patients in the control group could later switch to the PIM group, if PIM was prescribed, and could switch back again to the control group if the ‘washout’ phase was completed and no new PIM was prescribed.

### Statistical analysis

Continuous variables were described by median and interquartile range (IQR). Categorical data were described by absolute frequencies and percentages. The risk of ADE within the defined time horizon of 30 days after filling a prescription for a particular PIM was estimated as the proportion of patients with an occurrence of an ADE relative to the total number of patients receiving prescriptions for that PIM. Corresponding exact 95% confidence intervals (CIs) were estimated by the Clopper-Pearson method. We considered a risk of > 1% as an indicator for clinically relevant increase in risk of ADE.

To compare the risk of ADE between patients who filled a particular PIM (PIM group) and the control group, MSM [26] were constructed for each PIM included in the EU(7)-PIM list and AT-PIM list, respectively. These comparisons were all adjusted for time-dependent confounders where the dynamic covariates according to a patient’s 90-days medical history (comorbidities defined as ICD10-chapters based on hospital discharge diagnoses (12 binary variables), co-medication defined as ATC 2nd levels based on filled prescriptions (96 binary variables), and total number of days of hospitalization) and demographic data (current age and sex) were considered. First, propensity scores (PS) were estimated as the predicted probabilities of filling a prescription for a PIM modeled by logistic regression of the PIM status (PIM or control) on all the covariates listed above, where the variables age and hospitalization days were modelled as restricted cubic spline functions with 4 knots. Second, from the propensity scores inverse probability weights (IPW) were computed as 1/PS and 1/(1-PS) for patients with PIM and controls, respectively. By inverse probability weighting, the two groups were made comparable as if they constituted a pseudo-population which was randomized to PIM and control groups. The IPW were used to estimate weighted risks of ADE in both groups (PIM and control group) and the corresponding weighted relative risks and weighted risk differences. *p*-values for those quantities and standard errors for the computation of 95% CIs were obtained by blocked bootstrap, where patients were resampled 100-times with replacement such that all observations of that patient were simultaneously included in a resample if that patient was sampled. More information on this methodology can be found in the work of Hernan et al. [26]. *p*-values were corrected for the multiplicity by controlling the false discovery rate at 10%.

In addition to the analyses of substances listed as PIM in the EU(7)-PIM list and AT-PIM list respectively, we also screened other substances not included in the PIM lists for being potentially inappropriate for older CKD patients. By taking into account the 90-days washout-phase prior to a prescription, we first evaluated how often after filling a prescription for a substance an ADE followed within 30 days. Given the marginal incidences of ADE and prescriptions for that substance, we computed the expected distribution of such events under the null hypothesis that assumed no causal relationship between prescription and ADE. *p*-values were derived as the probabilities by which events were expected to randomly occur at least as often as observed. The p-values obtained were corrected for multiplicity by the procedure of Benjamini and Hochberg [27] which controls the false discovery rate. The number needed to harm, describing how many prescriptions were needed in order to expect one ADE, was calculated as the ratio between the total number of filled prescriptions for a substance and the number of filled prescriptions after which an ADE occurred.

Statistical analysis was performed using R software (version: 3.6.1) [28].

### Sample size calculation

Approximately 250,000 inhabitants of Lower Austria are of age 70 years or older [29]. Assuming a CKD prevalence of approximately 5% among them results in an estimated study population size of 12,500 people. If we assume a PIM utilization of 10%, and ADE risks of 1.5 and 0.5% in the PIM and control groups, then the analysis has approximately 90% power to find a significantly increased ADE risk among PIM users at a significance level of 5%. The expected width of a 95% confidence interval for the ADE risk among PIM users is 1.6 percentage points.

### Ethics

Data were anonymised to preserve patients’ privacy. Data storage and handling were in agreement with data protection laws. This study was supported by the Main Association of Austrian Social Insurance Institutions, approved by the Ethics Committee of the Medical University of Vienna (EK-No. 2278/2017) and performed in accordance with the Declaration of Helsinki. This was a retrospective study, therefore, informed consent was waived.

## Results

### Study population

We identified 11,547 patients who fulfilled the inclusion criteria and were eligible for the study. In 2008 patients were in median 78 (IQR: 73–83) years old, and 5845 (50.6%) were female. In total, 40,999 hospitalizations were recorded for this study population between 2008 and 2011. In this period patients had a median of 2 (IQR: 1–5) hospitalizations with a median hospital stay duration of 5 (IQR: 2–11) days per hospitalization. 4950 (43%) patients died during the follow-up between 2008 and 2011.

### Prescriptions of PIM

This population of patients filled a total of 2,401,434 prescriptions during the follow-up period. 593,783 (24.7%) and 193,809 (8.1%) of the prescriptions contained a medication with a substance listed in the EU(7)- and AT-PIM list, respectively. The prescriptions for 157 (47.6%) out of 330 different PIM included in the EU(7)-PIM list and for 65 (86.7%) out of 75 different PIM in the AT-PIM list were filled by the patients during the follow-up. The ten most frequently prescribed PIM are listed in Table 1. 10,925 (94.6%) and 9160 (79.3%) patients received at least one EU(7)-PIM and AT-PIM prescription, respectively. In median one patient filled 60 (IQR: 33–94) prescriptions per year; of these 15 (IQR: 7–25) prescriptions per year were from the EU(7)-PIM and 6 (IQR: 2–12) from the AT-PIM list, respectively. General practitioners prescribed 90.4% of all filled prescriptions, and 92.2% of the recorded PIM.

**Table 1:** Most frequently prescribed potentially inappropriate medication from the EU(7)-PIM and AT-PIM list (described by ATC code and name of the substance) together with the number of filled prescriptions between 2008 and 2011, patients’ median (IQR) age at the time of filling the prescription and number (%) of different patients filling the prescription

ATC code	Substance	PIM list	ADE, increased risk of	Prescriptions, n	Patients’ age (years), median (IQR)	Patients, n (%)
A02BC02	Pantoprazole	EU(7)	- *C. difficile* infection - hip fracture	107,233	79 (74–84)	6.947 (60.2)
A02BC03	Lansoprazole	EU(7)	- *C. difficile* infection - hip fracture	47,291	80 (75–85)	3.238 (28.0)
N06DX02	*Ginkgo biloba*	EU(7), AT	- orthostatic hypotension and fall	31,395	81 (77–86)	2.266 (19.6)
N02AX02	Tramadol	EU(7), AT	- CNS side effects	28,130	78 (74–84)	4.115 (35.6)
M01AB05	Diclofenac	EU(7), AT	- GI bleeding, ulceration, or perforation	23,001	78 (73–83)	4.399 (38.1)
A02BC01	Omeprazole	EU(7)	- *C. difficile* infection - hip fracture	21,601	79 (74–85)	1.657 (14.3)
A02BC05	Esomeprazole	EU(7)	- *C. difficile* infection - hip fracture	20,821	79 (74–84)	1.682 (14.6)
C02CA04	Doxazosin	EU(7)	- orthostatic hypotension - dry mouth - urinary incontinence/ impaired micturition - CNS side effects	18,704	77 (73–82)	1.133 (9.8)
C04AD03	Pentoxifyllin	EU(7), AT	- orthostatic hypotension and fall	12,658	80 (76–85)	1.065 (9.2)
C02AC06	Rilmenidine	EU(7)	- orthostatic hypotension - bradycardia - CNS side effects	12,241	78 (73–83)	919 (8.0)

*ATC code* Anatomical Therapeutic Chemical Classification System, *IQR* Interquartile range

### The risk of ADE after filling a prescription for PIM

In the data we could identify only few ADE that occurred within 30 days after a patient had filled a prescription for PIM. Therefore, the risks of ADE were small and did not exceed 1% for any of the filled PIM. For 7 PIM the upper confidence interval limit fell below 1%, hence, no clinically important increase in risk after filling a prescription for these PIM could be detected. For other PIM the results were less clear due to too few filled prescriptions. These results are summarized in Table 2.

**Table 2:** The risk of adverse drug events with 95% confidence intervals (CI) within 30 days after filling a prescription for potentially inappropriate medication (described by ATC code and name of the substance) listed in the EU(7)-PIM list or in the AT-PIM list along with observed frequencies of prescriptions and adverse drug events during the study period

ATC code	Substance	ADE, increased risk of	PIM list	Prescriptions, n	ADE, n	Risk of ADE % (95% CI)
A02BC02	Pantoprazole	- *C. difficile* infection - hip fracture	EU(7)	2162	4	0.19 (0.05, 0.47)
M01AB05	Diclofenac	- GI bleeding, ulceration, or perforation	EU(7), AT	1908	3	0.16 (0.03, 0.46)
N02AX02	Tramadol	- CNS side effects	EU(7), AT	1300	3	0.23 (0.05, 0.67)
C03DA01	Spironolactone	- hyperkalaemia - hyponatremia	EU(7)	871	2	0.23 (0.03, 0.83)
M01AG01	Mefenamic acid	- GI bleeding, ulceration, or perforation	EU(7)	757	4	0.53 (0.14, 1.35)
N06DX02	*Ginkgo biloba*	- orthostatic hypotension and fall	EU(7), AT	721	1	0.14 (0.00, 0.77)
A03FA01	Metoclopramid	- may worsen peripheral arterial blood flow and precipitate intermittent claudication	EU(7)	634	1	0.16 (0.00, 0.88)
A02BC03	Lansoprazole	- *C. difficile* infection - hip fracture	EU(7)	570	3	0.53 (0.11, 1.53)
C01AA04	Digitoxin	- elevated glycoside sensitivity - intoxication	EU(7)	512	3	0.57 (0.12, 1.70)
B01AA07	Acenocoumarol	- bleeding	EU(7)	480	1	0.21 (0.01, 1.16)
C01BD01	Amiodarone	- QT interval problems - torsades de pointes	EU(7)	438	0	0.00 (0.00, –0.84)
C01AA08	Metildigitoxin	- elevated glycoside sensitivity - intoxication	EU(7)	382	1	0.26 (0.01, 1.45)
G04BD09	Trospium	- anticholinergic side effects	EU(7)	366	0	0.00 (0.00, 1.00)
N05CD05	Triazolam	- falls and hip fracture - prolonged reaction time - psychiatric reactions	EU(7), AT	329	1	0.30 (0.01, 1.68)
N05BA12	Alprazolam	- falls and hip fracture - prolonged reaction time - psychiatric reactions	EU(7)	289	0	0.00 (0.00, 1.27)
N06AA09	Amitriptyline	- peripheral anticholinergic side effects - central anticholinergic side effects - cognitive deficit	EU(7), AT	280	0	0.00 (0.00, 1.31)
R03DA04	Theophylline	- CNS stimulant effects	EU(7), AT	275	0	0.00 (0.00, 1.33)
A02BC05	Esomeprazol	- *C. difficile* infection - hip fracture	EU(7)	271	0	0.00 (0.00, 1.35)
N05BA08	Bromazepam	- falls and hip fracture - prolonged reaction time - psychiatric reactions - cognitive impairment - depression	EU(7), AT	271	0	0.00 (0.00, 1.35)
N05AX08	Risperidone	- behavioural symptoms of dementia -increased mortality	AT	252	0	0.00 (0.00, 1.45)
C02CA04	Doxazosin	- orthostatic hypotension - dry mouth - urinary incontinence/ impaired micturition	EU(7)	242	0	0.00 (0.00, 1.51)
G03CA04	Estriol	- carcinogenic potential	EU(7)	232	1	0.43 (0.01, 2.38)
A02BC01	Omeprazole	- *C. difficile* infection - hip fracture	EU(7)	229	0	0.00 (0.00, 1.60)
C04AD03	Pentoxyfylline	- orthostatic hypotension and fall	EU(7), AT	208	0	0.00 (0.00, 1.76)

*ATC code* Anatomical Therapeutic Chemical Classification System, *ADE* Adverse drug event, *CI* Confidence interval

### The risk of ADE after filling a prescription for PIM as compared to the control group

In the following analysis we considered the 46 PIM that were filled for a minimum of 100 times. The results presented as weighted relative risks and weighted risk differences adjusted for covariates are shown in Table 3 for the substances where significant effects by controlling the false discovery rate at 10% were detected. The risk of ADE after filling PIM prescription for 11 substances was significantly increased as compared to the control group. These substances were omeprazole, pantoprazole, lansoprazole, esomeprazole, metoclopramide, digitoxin, rilmenidine, spironolactone, tramadol, oxazepam, and theophylline. The risk was significantly reduced for the following 4 substances: metildigoxin, carbamazepine, diazepam, and amitriptyline.

**Table 3:** Risk of ADE after filling a prescription for potentially inappropriate medication (described by ATC code and name of the substance) included in the EU(7)-PIM or AT-PIM list as compared to the control group described as relative risk and risk difference with 95% confidence intervals (CI), uncorrected *p*-values and *p*-values corrected for multiplicity by controlling the false discovery rate

ATC code	Substance name	PIM list	Relative risk (95% CI)	Risk difference (95% CI)	***P***-value	Corrected ***p***-value
A02BC01	Omeprazole	EU(7)	2.95 (1.3, 6.73)	0.02 (0.01, 0.03)	0.010	0.033
A02BC02	Pantoprazole	EU(7)	3.71 (1.37, 10.05)	0.03 (0.02, 0.03)	0.010	0.033
A02BC03	Lansoprazole	EU(7)	1.87 (1.16, 3.01)	0.01 (0, 0.02)	0.010	0.033
A02BC05	Esomeprazole	EU(7)	3.87 (1.38, 10.85)	0.03 (0.02, 0.04)	0.010	0.033
A03FA01	Metoclopramide	EU(7)	2.44 (1.24, 4.79)	0.02 (0.01, 0.02)	0.010	0.033
C01AA04	Digitoxin	EU(7)	1.89 (1.17, 3.06)	0.01 (0, 0.02)	0.010	0.033
C01AA08	Metildigoxin	EU(7)	0.37 (0.17, 0.79)	−0.01 (−0.01, 0)	0.010	0.033
C02AC06	Rilmenidine	EU(7)	2.35 (1.09, 5.09)	0.01 (0, 0.03)	0.030	0.094
C03DA01	Spironolactone	EU(7)	2.47 (1.24, 4.91)	0.02 (0.01, –0.02)	0.010	0.033
N02AX02	Tramadol	EU(7), AT	2.46 (1.24, –4.89)	0.02 (0.01, 0.02)	0.010	0.033
N03AF01	Carbamazepine	EU(7)	0 (0, NaN)	−0.01 (−0.01, −0.01)	0.010	0.033
N05BA01	Diazepam	EU(7), AT	0.4 (0.2, 0.8)	−0.01 (− 0.01, 0)	0.010	0.033
N05BA04	Oxazepam	EU(7), AT	2.85 (1.11, 7.33)	0.02 (0, 0.04)	0.030	0.094
N06AA09	Amitriptyline	EU(7), AT	0.42 (0.22, 0.81)	–0.01 (−0.01, 0)	0.010	0.033
R03DA04	Theophylline	EU(7), AT	2.32 (1.22, –4.41)	0.01 (0.01, 0.02)	0.010	0.033

*ATC code* Anatomical Therapeutic Chemical Classification System

### Explorative analysis of new PIM

In addition to the analysis of substances that are already included in the PIM lists we also investigated whether any other substance increased the risk of ADE. An excerpt of these results is reported in Table 4. For clarity, we limit the findings only to substances where the strength of association resulted in an uncorrected *p*-value smaller than 10%. Several antibiotic medicines, some pulmonary medicine and one anticoagulant showed a significant increase in risk of ADE. These associations were, however, not statistically significant after correcting for multiplicity (Table 4).

**Table 4:** Explorative analysis of (un)listed potentially inappropriate medication (described by ATC code and name of the substance) for older adults with chronic kidney disease. For each medication the total number of prescriptions and adverse drug events, a number needed to harm, an uncorrected p-value and a p-value corrected for the multiplicity by controlling the false discovery rate are presented. Of these medication midazolam, ofloxacin and dabigatran etexilate are included in the EU(7)-PIM list

ATC code	Substance name	Prescriptions, n	ADE, n	NNH	***P***-value	Corrected ***p***-value
N05CD08	Midazolam	2	1	2	< 0.001	0.118
J01XA01	Vancomycin	5	1	5	0.001	0.450
C10AC01	Cholestyramine	65	3	21.7	0.004	0.749
J02AC01	Fluconazole	219	6	36.5	0.007	0.829
N05AL05	Amisulpride	47	2	23.5	0.012	0.892
N06AG02	Moclobemide	17	1	17	0.013	0.897
R05DB05	Pentoxyverine	21	1	21	0.019	0.929
M01AG	Fenamate	24	1	24	0.024	0.943
V03AE03	Lanthanum carbonate	168	4	42	0.028	0.950
D01AC12	Fenticonazole	65	2	32.5	0.028	0.950
J01CF05	Flucloxacillin	27	1	27	0.030	0.954
J01CR04	Sultamicillin	181	4	45.3	0.036	0.961
J01EA01	Trimethoprim	747	12	62.3	0.040	0.965
L01AA01	Cyclophosphamide	32	1	32	0.041	0.966
D01AE22	Naftifine	76	2	38	0.041	0.966
D01AE15	Terbinafine	34	1	34	0.046	0.969
C09DX03	Olmesartan medoxomil, Amlodipine and Hydrochlorothiazide	36	1	36	0.051	0.972
P01AB01	Metronidazole	341	6	56.8	0.057	0.975
C05AX03	Haemorrhoid treatment	40	1	40	0.061	0.977
N02AA59	Codeine	41	1	41	0.064	0.978
J01MA01	Ofloxacin	301	5	60.2	0.083	0.983
B01AE07	Dabigatran etexilate	103	2	51.5	0.085	0.983
G01AX11	Povidone-iodine	50	1	50	0.089	0.984
R03BB01	Ipratropium bromide	110	2	55	0.098	0.985

*ATC code* Anatomical Therapeutic Chemical Classification System, *ADE* Adverse drug event, *NNH* Number needed to harm

## Discussion

In this retrospective epidemiological cohort study we estimated utilization statistics of PIM in patients with CKD aged 70 years or more and estimated the risk of ADE after a prescription of PIM was filled. We observed that 65 of the 75 (86.7%) medications that were suggested as potentially inappropriate for older adults in Austria were prescribed to those patients. This number is substantially higher than reported results from a previous study conducted in geriatric patients in Austria [10], and is comparable to the numbers reported in previous studies from Europe, Australia, and the US [13, 14, 30, 31]. A gender-specific difference in the prescription of PIM could not be detected in our study.

The utilization of PIM in Austria has been shifted from non-steroidal anti-inflammatory drugs, vasodilators and psychotropic drugs to proton pump inhibitors (PPI) and *Ginkgo biloba* [11]. In Italy, ketorolac, amiodarone, and clonidine were the most prescribed PIM [32]. In Sweden, PIMs such as NSAIDs, hypnotic and sedative drugs, and apixaban were commonly prescribed [33]. One should note that results from previous studies conducted worldwide vary due to the variations in methods, data collection, differently defined study population, and patient characteristics [34, 35].

The fact that PPI are frequently used by older adults should cause some concern. It has been shown that older adults suffer more often from falls, fractures, and from Clostridium difficile-associated diarrhea [36–38]. The higher risk of ADE as compared to the control group, such as bleeding, ulcer, intoxication, neurological and psychological symptoms, after PPI-intake reported in the present study is in line with results from previous studies [39–46]. Alarming is that approximately 40% of older patients with PPI had no indication for PPI use [47]. Since the utilization of PPI has increased worldwide the indication for the prescription of PPI should be strictly examined.

Another major concern is the prescription of *Ginkgo biloba*. Some positive impact on cognitive impairment and Alzheimer’s disease is associated with its use, however, according to the EU(7)-PIM and AT-PIM list there is no efficacy proven for this herbal medicine. Moreover, it has been claimed that *Ginkgo biloba* is associated with increased risk of orthostatic hypotension and falls as well as increased cancer incidences for breast and colon [10, 48].

Our explorative analysis revealed several additional medications not yet included in the PIM lists that should be considered in the future research of PIM. Several antibiotics could be potentially harmful to geriatric patients with CKD. Although an increased risk of ADE could not be statistically confirmed after correcting for multiplicity, our results do not explicitly exclude this possibility because of an inflated type II error after multiplicity correction caused by rare occurrence of ADE and prescriptions for PIM. This is in accordance with results from a previous study that suggested vancomycin might cause nephropathy [49]. Fluconazole is prescribed for antifungal treatment of coccidioidomycosis, and it has already been reported that long-term use of fluconazole can lead to ADE in the population at risk [50]. Moreover, fluoroquinolone has been shown to cause ADE of central nervous system and tendinopathy in the older adults [51].

In the present study rilmenidine was found to cause an increased risk of ADE compared to the control group. This antihypertensive drug has been previously shown to be associated with an elevated risk for hospitalization and ADE in older adults [52]. In addition, patients receiving cardiovascular PIM such as doxazosin and rilmenidine, which had the highest prescribing frequency among cardiovascular PIM in the present study, have an increased risk of orthostatic hypotension [53, 54].

General practitioners were responsible for the majority of PIM prescriptions as they write most of all prescriptions in Austria. Patients usually receive their first prescription after being discharged from the hospital from their family doctor (general practitioner) or their specialist. The follow-up prescriptions are taken over by general practitioners.

The strengths of our study are well-defined inclusion criteria where only patients of at least 70 years with a chronic condition, i.e. CKD were selected. In such a population we can expect overall higher utilization of medication and possible changes in pharmacokinetics due to reduced kidney function causing accumulation of the drugs in the body that leads to likely toxicity and ADE. Furthermore, by using the scientific data base GAP-DRG2 that contains curated and cleaned data from a relatively large region of Lower Austria, which is Austria’s second most populous province, structural distortion of the results due to self-selection bias or poor linkage and data quality could be avoided. Nonetheless, by appropriate adjustment for time-dependent confounding using state-of-the-art methodology for causal inference, we avoided bias in estimates of risks of ADE in patients receiving PIM relative to the control group.

Our study has several limitations. By using claims data from only one region of Austria and restricting the study cohort to CKD patients, only 11,547 individuals were eligible for the study, the number that is rather low for the investigation of extremely rare events such as ADE. Therefore, no definite results that would clearly indicate exceedance of the threshold level of 1% were obtained from the analysis of risks of ADE within 30 days after filling the prescription for PIM. Another possible explanation for small numbers of ADE is that ICD10 codes could underestimate the true numbers. Also many minor ADE may have been handled in an outpatient setting and thus excluded from analyses. Moreover, while ICD10 codes of hospital discharge diagnosis were used to identify CKD patients, neither clinical diagnosis nor case severity was adjudicated for in the analysis. There also remains a possibility of information bias regarding the diagnoses registered for hospitalizations defining ADE. The patients that were eligible for the study were multimorbid and had a high risk of being exposed to polypharmacy. Therefore, to relate ADE to a particular PIM with a greater certainty, a criterion was set by which the investigation was limited to ADE that occurred due to incident drug therapy only, disregarding the chance that some ADE might have also occurred as a cause of prevalent drug therapy. With this strict criterion we could attribute ADE to newly prescribed PIM very specifically and avoiding false positive attributions but at the cost of probably even underestimating the ADE risk after PIM use. Information of prescribed medication was limited to those medicines which were reimbursed by health care providers, and over-the-counter medication was not included in our analysis. Nonetheless, we were not able to assess actual adherence to medication for which prescriptions had been filled. Lastly, by making use of a curated scientific database we had to compromise on the recency of the data. There is a risk that utilization may have changed within the last few years, and this may affect our estimated absolute risks and risk differences, but estimated relative risks, which are independent of the level of utilization of a PIM, are robust to potential trends in utilization.

## Conclusion

The awareness of PIM leading to ADE might be increased by PIM lists, however, drugs assigned as potentially inappropriate are not necessarily contraindicated. A population-wide study should be planned to record the usage of PIM among older adults in Austria and its consequences, e.g. the occurrence of ADE. Many substances are listed in both the EU(7)-PIM and the AT-PIM list, but only a small number of drugs could be linked to serious clinically relevant ADE due to incident therapy. To facilitate treatment of older adults that are often multimorbid, it is therefore advisable to consider adapting the AT-PIM list by adding the assessments of ADE severity by using a warning system similar to a traffic light system based on their severity score.

## Supplementary Information

**Additional file 1.** List of adverse drug events.

## Data Availability

The data that support the findings of this study are available from the Main Association of Austrian Social Security Institutions but restrictions apply to the availability of these data, which were used under license for the current study, and so are not publicly available. Data are however available from the authors upon reasonable request and with permission of the Main Association of Austrian Social Security Institutions.

## Abbreviations

ADE: Adverse drug events
ATC code: Anatomical Therapeutic Chemical Classification System
CI: Confidence intervals
CKD: Chronic kidney disease
ICD10: The 10th revision of the International Statistical Classification of Diseases and Related Health Problems
IPW: Inverse probability weights
IQR: Interquartile range
MSM: Marginal structural models
NSAID: Nonsteroidal anti-inflammatory drugs
PIM: Potentially inappropriate medication
PPI: Proton pump inhibitors
PS: Propensity score
