# Supplementary material for: Utilization of potentially inappropriate medication and risk of adverse drug events among older adults with chronic renal insufficiency: a population-wide cohort study
Source: BMC Geriatr. 2021 Feb 10;21:117. doi: 10.1186/s12877-021-02057-5 (PMC7877037; doi:10.1186/s12877-021-02057-5)
Supplement: Supplementary file 1 — Additional file 1. List of adverse drug events. [file 12877_2021_2057_MOESM1_ESM.pdf]

Utilization of potentially inappropriate medication and risk of adverse drug events among older adults with chronic renal insufficiency: a population-wide cohort study

Safoura Sheikh Rezaei, Hana Šinkovec, Alexander Schöberl, Christoph Rinner, Georg Heinze, Michael Wolzt, Walter Gall

Supplementary material

List of adverse drug events

List describing ICD10 codes indicating possible clinically relevant adverse drug events (ADE), sorted by categories defined in Stausberg and Hasford (2010) (A.1: a drug-related causation was noted in the ICD10; A.2: a drug- or other substance-related causation was noted in the ICD10; B.1: the event was denoted as a drug poisoning, thus implying an unphysiological dosage; B.2: the event was denoted as poisoning by or harmful use of drugs or other substances; C: a drug related causation was very likely; D: a drug-related causation was likely; E: a drug-related causation was possible). Codes within each category sorted by alphabetical order.

Category	ICD10	Description
A.1	D52.1	Drug-induced folate deficiency anaemia
A.1	D59.0	Drug-induced autoimmune haemolytic anaemia
A.1	D59.2	Drug-induced nonautoimmune haemolytic anaemia
A.1	D61.1	Transient acquired pure red cell aplasia/Other aplastic anaemias
A.1	D61.10	Transient acquired pure red cell aplasia
A.1	D61.18	Other aplastic anaemias
A.1	D61.19	Other aplastic anaemias, not specified
A.1	D69.52	Heparin induced thrombocytopenia Type I
A.1	D69.53	Heparin induced thrombocytopenia Type II
A.1	D70.1	Drug-induced agranulocytosis and neutropenia
A.1	D70.10	Drug-induced agranulocytosis and neutropenia; critical phase <4 days
A.1	D70.11	Drug-induced agranulocytosis and neutropenia; critical phase between 10 and 20 days
A.1	D70.12	Drug-induced agranulocytosis and neutropenia; critical phase >20 days
A.1	D70.18	Other disorders of agranulocytosis and neutropenia
A.1	D70.19	Drug-induced agranulocytosis and neutropenia, unspecified
A.1	E06.4	Drug-induced thyroiditis
A.1	E16.0	Drug-induced hypoglycaemia without coma
A.1	E23.1	Drug-induced hypopituitarism
A.1	E24.2	Drug-induced Cushing syndrome
A.1	E27.3	Drug-induced adrenocortical insufficiency
A.1	E66.1	Drug-induced obesity
A.1	E66.10	Drug-induced obesity: Body-Mass-Index [BMI] between 30 and 35
A.1	E66.11	Drug-induced obesity: Body-Mass-Index [BMI] between 35 and 40
A.1	E66.12	Drug-induced obesity: Body-Mass-Index [BMI] between 40 and 55
A.1	E66.19	Drug-induced obesity: Body-Mass-Index [BMI] unspecified
A.1	G21.0	Malignant neuroleptic syndrome
A.1	G21.1	Other drug-induced secondary parkinsonism
A.1	G24.0	Drug-induced dystonia
A.1	G25.1	Drug-induced tremor
A.1	G25.4	Drug-induced chorea

A.1	G25.6	Drug-induced tics and other tics of organic origin
A.1	G44.4	Drug-induced headache, not elsewhere classified
A.1	G62.0	Drug-induced polyneuropathy
A.1	G72.0	Drug-induced myopathy
A.1	H26.3	Drug-induced cataract
A.1	H40.6	Glaucoma secondary to drugs
A.1	I95.2	Hypotension due to drugs
A.1	J70.2	cute drug-induced interstitial lung disorders
A.1	J70.3	Chronic drug-induced interstitial lung disorders
A.1	J70.4	Drug-induced interstitial lung disorders, unspecified
A.1	K85.3	Drug-induced acute pancreatitis
A.1	K85.30	Drug-induced acute pancreatitis: without specifications on organ complication
A.1	K85.31	Drug-induced acute pancreatitis: with organ complication
A.1	L10.5	Drug-induced pemphigus
A.1	L43.2	Lichenoid drug reaction
A.1	L56.0	Drug phototoxic response
A.1	L56.1	Drug photoallergic response
A.1	L64.0	Drug-induced androgenic alopecia
A.1	M10.2	Drug-induced gout
A.1	M10.20	Drug-induced gout: multiple body regions
A.1	M10.21	Drug-induced gout: shoulder region
A.1	M10.22	Drug-induced gout: upper arm
A.1	M10.23	Drug-induced gout: forearm
A.1	M10.24	Drug-induced gout: hand
A.1	M10.25	Drug-induced gout: pelvic region and upper leg
A.1	M10.26	Drug-induced gout: lower leg
A.1	M10.27	Drug-induced gout: ankle and foot
A.1	M10.28	Drug-induced gout: other regions
A.1	M10.29	Drug-induced gout: unspecified regions
A.1	M32.0	Drug-induced systemic lupus erythematosus
A.1	M80.4	Drug-induced osteoporosis with pathological fracture
A.1	M80.40	Drug-induced osteoporosis with pathological fracture: multiple body regions
A.1	M80.41	Drug-induced osteoporosis with pathological fracture: shoulder region
A.1	M80.42	Drug-induced osteoporosis with pathological fracture: upper arm
A.1	M80.43	Drug-induced osteoporosis with pathological fracture: forearm
A.1	M80.44	Drug-induced osteoporosis with pathological fracture: hand
A.1	M80.45	Drug-induced osteoporosis with pathological fracture: pelvic region and upper leg
A.1	M80.46	Drug-induced osteoporosis with pathological fracture: lower leg
A.1	M80.47	Drug-induced osteoporosis with pathological fracture: ankle and foot
A.1	M80.48	Drug-induced osteoporosis with pathological fracture: other regions
A.1	M80.49	Drug-induced osteoporosis with pathological fracture: unspecified regions
A.1	M81.4	Drug-induced osteoporosis
A.1	M81.40	Drug-induced osteoporosis: multiple body regions
A.1	M81.41	Drug-induced osteoporosis: shoulder region
A.1	M81.42	Drug-induced osteoporosis: upper arm
A.1	M81.43	Drug-induced osteoporosis: forearm
A.1	M81.44	Drug-induced osteoporosis: hand

A.1	M81.45	Drug-induced osteoporosis: pelvic region and upper leg
A.1	M81.46	Drug-induced osteoporosis: lower leg
A.1	M81.47	Drug-induced osteoporosis: ankle and foot
A.1	M81.48	Drug-induced osteoporosis: other regions
A.1	M81.49	Drug-induced osteoporosis: unspecified regions
A.1	M83.5	Other drug-induced osteomalacia in adults
A.1	M83.50	Other drug-induced osteomalacia in adults: multiple regions
A.1	M83.51	Other drug-induced osteomalacia in adults: shoulder regions
A.1	M83.52	Other drug-induced osteomalacia in adults: upper arm
A.1	M83.53	Other drug-induced osteomalacia in adults: forearm
A.1	M83.54	Other drug-induced osteomalacia in adults: hand
A.1	M83.55	Other drug-induced osteomalacia in adults: pelvic region and upper leg
A.1	M83.56	Other drug-induced osteomalacia in adults: lower leg
A.1	M83.57	Other drug-induced osteomalacia in adults: ankle and foot
A.1	M83.58	Other drug-induced osteomalacia in adults: other regions
A.1	M83.59	Other drug-induced osteomalacia in adults: unspecified regions
A.1	M87.1	Osteonecrosis due to drugs
A.1	M87.10	Osteonecrosis due to drugs: multiple regions
A.1	M87.11	Osteonecrosis due to drugs: shoulder region
A.1	M87.12	Osteonecrosis due to drugs: upper arm
A.1	M87.13	Osteonecrosis due to drugs: forearm
A.1	M87.14	Osteonecrosis due to drugs: hand
A.1	M87.15	Osteonecrosis due to drugs: pelvic region and upper leg
A.1	M87.16	Osteonecrosis due to drugs: lower leg
A.1	M87.17	Osteonecrosis due to drugs: ankle and foot
A.1	M87.18	Osteonecrosis due to drugs: other regions
A.1	M87.19	Osteonecrosis due to drugs: unspecified regions
A.1	N14.0	Analgesic nephropathy
A.1	O74.4	Toxic reaction to local anaesthesia during labour and delivery
A.1	R50.2	Drug-induced fever
A.1	T88.3	Malignant hyperthermia due to anaesthesia
A.2	D64.2	Secondary sideroblastic anaemia due to drugs and toxins
A.2	E03.2	Hypothyroidism due to medicaments and other exogenous substances
A.2	F11	Mental and behavioural disorders due to use of opioids
A.2	F11.0	Mental and behavioural disorders due to use of opioids: acute intoxication
A.2	F11.1	Mental and behavioural disorders due to use of opioids: harmful use
A.2	F11.2	Mental and behavioural disorders due to use of opioids: dependence syndrome
A.2	F11.3	Mental and behavioural disorders due to use of opioids: withdrawal state
A.2	F11.4	Mental and behavioural disorders due to use of opioids: withdrawal state with delirium
A.2	F11.5	Mental and behavioural disorders due to use of opioids: psychotic disorder
A.2	F11.6	Mental and behavioural disorders due to use of opioids: amnestic syndrome
A.2	F11.7	Mental and behavioural disorders due to use of opioids: residual and late-onset psychotic disorder
A.2	F11.8	Mental and behavioural disorders due to use of opioids: other mental and behavioural disorders
A.2	F11.9	Mental and behavioural disorders due to use of opioids: unspecified mental and behavioural disorders
A.2	F13	Mental and behavioural disorders due to use of sedatives or hypnotics
A.2	F13.0	Mental and behavioural disorders due to use of sedatives or hypnotics: acute intoxication
A.2	F13.1	Mental and behavioural disorders due to use of sedatives or hypnotics: harmful use

A.2	F13.2	Mental and behavioural disorders due to use of sedatives or hypnotics: dependence syndrome
A.2	F13.3	Mental and behavioural disorders due to use of sedatives or hypnotics: withdrawal state
A.2	F13.4	Mental and behavioural disorders due to use of sedatives or hypnotics: withdrawal state with delirium
A.2	F13.5	Mental and behavioural disorders due to use of sedatives or hypnotics: psychotic disorder
A.2	F13.6	Mental and behavioural disorders due to use of sedatives or hypnotics: amnestic syndrome
A.2	F13.7	Mental and behavioural disorders due to use of sedatives or hypnotics: residual and late-onset psychotic disorder
A.2	F13.8	Mental and behavioural disorders due to use of sedatives or hypnotics: other mental and behavioural disorders
A.2	F13.9	Mental and behavioural disorders due to use of sedatives or hypnotics: unspecified mental and behavioural disorders
A.2	F15	Mental and behavioural disorders due to use of other stimulants, including caffeine
A.2	F15.0	Mental and behavioural disorders due to use of other stimulants, including caffeine: acute intoxication
A.2	F15.1	Mental and behavioural disorders due to use of other stimulants, including caffeine: harmful use
A.2	F15.2	Mental and behavioural disorders due to use of other stimulants, including caffeine: dependence syndrome
A.2	F15.3	Mental and behavioural disorders due to use of other stimulants, including caffeine: withdrawal state
A.2	F15.4	Mental and behavioural disorders due to use of other stimulants, including caffeine: withdrawal state with delirium
A.2	F15.5	Mental and behavioural disorders due to use of other stimulants, including caffeine: psychotic disorder
A.2	F15.6	Mental and behavioural disorders due to use of other stimulants, including caffeine: amnestic disorder
A.2	F15.7	Mental and behavioural disorders due to use of other stimulants, including caffeine: residual and late-onset psychotic disorder
A.2	F15.8	Mental and behavioural disorders due to use of other stimulants, including caffeine: other mental and behavioural disorders
A.2	F15.9	Mental and behavioural disorders due to use of other stimulants, including caffeine: unspecified mental and behavioural disorders
A.2	F19	Mental and behavioural disorders due to multiple drug use and use of other psychoactive substances
A.2	F19.0	Mental and behavioural disorders due to multiple drug use and use of other psychoactive substances: acute intoxication
A.2	F19.1	Mental and behavioural disorders due to multiple drug use and use of other psychoactive substances: harmful use
A.2	F19.2	Mental and behavioural disorders due to multiple drug use and use of other psychoactive substances: dependence syndrome
A.2	F19.3	Mental and behavioural disorders due to multiple drug use and use of other psychoactive substances: withdrawal state
A.2	F19.4	Mental and behavioural disorders due to multiple drug use and use of other psychoactive substances: withdrawal state with delirium
A.2	F19.5	Mental and behavioural disorders due to multiple drug use and use of other psychoactive substances:psychotic disorder
A.2	F19.6	Mental and behavioural disorders due to multiple drug use and use of other psychoactive substances: amnestic disorder
A.2	F19.7	Mental and behavioural disorders due to multiple drug use and use of other psychoactive substances: residual and late-onset psychotic disorder
A.2	F19.8	Mental and behavioural disorders due to multiple drug use and use of other psychoactive substances: other mental and behavioural disorders
A.2	F19.9	Mental and behavioural disorders due to multiple drug use and use of other psychoactive substances: unspecified mental and behavioural disorders
A.2	F52	Sexual dysfunction, not caused by organic disorder or disease
A.2	G21.2	Secondary parkinsonism due to other external agents
A.2	I42.7	Cardiomyopathy due to drugs and other external agents
A.2	L23.3	Allergic contact dermatitis due to drugs in contact with skin
A.2	L24.4	Irritant contact dermatitis due to drugs in contact with skin
A.2	L25.1	Unspecified contact dermatitis due to drugs in contact with skin
A.2	L27.0	Generalized skin eruption due to drugs and medicaments
A.2	L27.1	Localized skin eruption due to drugs and medicaments
A.2	L27.8	Dermatitis due to other substances taken internally
A.2	L27.9	Dermatitis due to unspecified substance taken internally
A.2	M34.2	Systemic sclerosis induced by drugs and chemicals
A.2	N14	Drug- and heavy-metal-induced tubulo-interstitial and tubular conditions
A.2	N14.1	Nephropathy induced by other drugs, medicaments and biological substances

A.2	N14.2	Nephropathy induced by unspecified drug, medicament or biological substance
A.2	N14.3	Nephropathy induced by heavy metals
A.2	N14.4	Toxic nephropathy, not elsewhere classified
A.2	T78.2	Anaphylactic shock, unspecified
A.2	T78.3	Angioneurotic oedema
A.2	T78.4	Allergy, unspecified
A.2	T78.8	Other adverse effects, not elsewhere classified
A.2	T78.9	Adverse effect, unspecified
A.2	T80	Complications following infusion, transfusion and therapeutic injection
A.2	T80.1	Vascular complications following infusion, transfusion and therapeutic injection
A.2	T80.2	Infections following infusion, transfusion and therapeutic injection
A.2	T80.3	ABO incompatibility reaction
A.2	T80.4	Rh incompatibility reaction
A.2	T80.5	Anaphylactic shock due to serum
A.2	T80.6	Other serum reactions
A.2	T80.8	Other complications following infusion, transfusion and therapeutic injection
A.2	T80.9	Unspecified complication following infusion, transfusion and therapeutic injection
A.2	T88.6	Anaphylactic shock due to adverse effect of correct drug or medicament properly administered
A.2	T88.7	Unspecified adverse effect of drug or medicament
A.2	Y57	Other and unspecified drugs and medicaments
A.2	Y57.9	Drug or medicament, unspecified
A.2	Y59	Other and unspecified vaccines and biological substances
A.2	Y59.9	Vaccine or biological substance, unspecified
B.1	T36	Poisoning by systemic antibiotics
B.1	T36.0	Poisoning by systemic antibiotics: Penicillin
B.1	T36.1	Poisoning by systemic antibiotics: Cefalosporins and other beta-lactam antibiotics
B.1	T36.2	Poisoning by systemic antibiotics: Chloramphenicol group
B.1	T36.3	Poisoning by systemic antibiotics: Marcolides
B.1	T36.4	Poisoning by systemic antibiotics: Tetracyclines
B.1	T36.5	Poisoning by systemic antibiotics: Aminoglycosides
B.1	T36.6	Poisoning by systemic antibiotics: Rifamycins
B.1	T36.7	Poisoning by systemic antibiotics: Antifungal antibiotics, systemically used
B.1	T36.8	Poisoning by systemic antibiotics: other systemic antibiotics
B.1	T36.9	Poisoning by systemic antibiotics: systemic antibiotics, unspecified
B.1	T37	Poisoning by other systemic anti-infectives and antiparasitics
B.1	T37.0	Poisoning by other systemic anti-infectives and antiparasitics: Sulfonamides
B.1	T37.1	Poisoning by other systemic anti-infectives and antiparasitics: Antimycobacterial drugs
B.1	T37.2	Poisoning by other systemic anti-infectives and antiparasitics: Antimalarials and drugs acting on other blood protozoa
B.1	T37.3	Poisoning by other systemic anti-infectives and antiparasitics: Other antiprotozoal drugs
B.1	T37.4	Poisoning by other systemic anti-infectives and antiparasitics: Anthelminthics
B.1	T37.5	Poisoning by other systemic anti-infectives and antiparasitics: Antiviral drugs
B.1	T37.8	Poisoning by other systemic anti-infectives and antiparasitics: Other specified systemic anti-infectives and antiparasitics
B.1	T37.9	Poisoning by other systemic anti-infectives and antiparasitics: Systemic anti-infective and antiparasitics, unspecified
B.1	T38	Poisoning by hormones and their synthetic substitutes and antagonists, not elsewhere classified
B.1	T38.0	Poisoning by hormones and their synthetic substitutes and antagonists, not elsewhere classified: Glucocorticoids and synthetic analogues
B.1	T38.1	Poisoning by hormones and their synthetic substitutes and antagonists, not elsewhere classified: Thyroid hormones and substitutes

B.1	T38.2	Poisoning by hormones and their synthetic substitutes and antagonists, not elsewhere classified: Antithyroid drugs
B.1	T38.3	Poisoning by hormones and their synthetic substitutes and antagonists, not elsewhere classified: Insulin and oral hypoglycaemic drugs
B.1	T38.4	Poisoning by hormones and their synthetic substitutes and antagonists, not elsewhere classified: Oral contraceptives
B.1	T38.5	Poisoning by hormones and their synthetic substitutes and antagonists, not elsewhere classified: other estrogens and progestogens
B.1	T38.6	Poisoning by hormones and their synthetic substitutes and antagonists, not elsewhere classified: Antigonadotrophins, antiestragens, antiandrogens, not elsewhere classified
B.1	T38.7	Poisoning by hormones and their synthetic substitutes and antagonists, not elsewhere classified: Androgens and anabolic congeners
B.1	T38.8	Poisoning by hormones and their synthetic substitutes and antagonists, not elsewhere classified: Other and unspecified hormones and their synthetic substitutes
B.1	T38.9	Poisoning by hormones and their synthetic substitutes and antagonists, not elsewhere classified: other and unspecified hormone antagonists
B.1	T39	Poisoning by nonopioid analgesics, antipyretics and antirheumatics
B.1	T39.0	Poisoning by nonopioid analgesics, antipyretics and antirheumatics: Salicylates
B.1	T39.1	Poisoning by nonopioid analgesics, antipyretics and antirheumatics: 4-Aminophenol derivates
B.1	T39.2	Poisoning by nonopioid analgesics, antipyretics and antirheumatics: Pyrazolone derivates
B.1	T39.3	Poisoning by nonopioid analgesics, antipyretics and antirheumatics: Other nonsteroidal anti-inflammatory drugs [NSAID]
B.1	T39.4	Poisoning by nonopioid analgesics, antipyretics and antirheumatics: Antirheumatics, not elsewhere classified
B.1	T39.8	Poisoning by nonopioid analgesics, antipyretics and antirheumatics: Other nonopioid analgesics and antipyretics, not elsewhere classified
B.1	T39.9	Poisoning by nonopioid analgesics, antipyretics and antirheumatics: Nonopoid analgetic, antipyretic and antirheumatic, unspecified
B.1	T40	Poisoning by narcotics and psychodysleptics [hallucinogens]
B.1	T40.0	Poisoning by narcotics and psychodysleptics [hallucinogens]: Opium
B.1	T40.1	Poisoning by narcotics and psychodysleptics [hallucinogens]: Heroin
B.1	T40.2	Poisoning by narcotics and psychodysleptics [hallucinogens]: Other opioids
B.1	T40.3	Poisoning by narcotics and psychodysleptics [hallucinogens]: Methadone
B.1	T40.4	Poisoning by narcotics and psychodysleptics [hallucinogens]: Other synthetic narcotics
B.1	T40.5	Poisoning by narcotics and psychodysleptics [hallucinogens]: Cocaine
B.1	T40.6	Poisoning by narcotics and psychodysleptics [hallucinogens]: Other and unspecified narcotics
B.1	T40.7	Poisoning by narcotics and psychodysleptics [hallucinogens]: Cannabis (derivates)
B.1	T40.8	Poisoning by narcotics and psychodysleptics [hallucinogens]: Lysergide (LSD)
B.1	T40.9	Poisoning by narcotics and psychodysleptics [hallucinogens]: Other and unspecified psychodysleptics [hallucinogens]
B.1	T41	Poisoning by anaesthetics and therapeutic gases
B.1	T41.0	Poisoning by anaesthetics and therapeutic gases: Inhaled anaesthetics
B.1	T41.1	Poisoning by anaesthetics and therapeutic gases: Intravenous anaesthetics
B.1	T41.2	Poisoning by anaesthetics and therapeutic gases: Other and unspecified general anaesthetics
B.1	T41.3	Poisoning by anaesthetics and therapeutic gases: Local Anaesthetics
B.1	T41.4	Poisoning by anaesthetics and therapeutic gases: Anaesthetics, unspecified
B.1	T41.5	Poisoning by anaesthetics and therapeutic gases: Therapeutic gases
B.1	T42	Poisoning by antiepileptic, sedative-hypnotic and antiparkinsonism drugs
B.1	T42.0	Poisoning by antiepileptic, sedative-hypnotic and antiparkinsonism drugs: Hydantoin derivates
B.1	T42.1	Poisoning by antiepileptic, sedative-hypnotic and antiparkinsonism drugs: Iminostilbenes
B.1	T42.2	Poisoning by antiepileptic, sedative-hypnotic and antiparkinsonism drugs: Succinimides and Oxazolidinediones
B.1	T42.3	Poisoning by antiepileptic, sedative-hypnotic and antiparkinsonism drugs: Barbiturates
B.1	T42.4	Poisoning by antiepileptic, sedative-hypnotic and antiparkinsonism drugs: Benzidiazepines
B.1	T42.5	Poisoning by antiepileptic, sedative-hypnotic and antiparkinsonism drugs: Mixed antiepileptics, not elsewhere classified
B.1	T42.6	Poisoning by antiepileptic, sedative-hypnotic and antiparkinsonism drugs: Other antiepileptic and sedative-hypnotic drugs
B.1	T42.7	Poisoning by antiepileptic, sedative-hypnotic and antiparkinsonism drugs: Antiepileptic and sedative-hypnotic drugs, unspecified

B.1	T42.8	Poisoning by antiepileptic, sedative-hypnotic and antiparkinsonism drugs: Antiparkinsonism drugs and other central muscle-tone depressants
B.1	T43	Poisoning by psychotropic drugs, not elsewhere classified
B.1	T43.0	Poisoning by psychotropic drugs, not elsewhere classified: Tricyclic and tetracyclic antidepressants
B.1	T43.1	Poisoning by psychotropic drugs, not elsewhere classified: Monoamine-oxidase-inhibitor antidepressants
B.1	T43.2	Poisoning by psychotropic drugs, not elsewhere classified: Other and unspecified antidepressants
B.1	T43.3	Poisoning by psychotropic drugs, not elsewhere classified: Phenothiazine antipsychotics and neuroleptics
B.1	T43.4	Poisoning by psychotropic drugs, not elsewhere classified: Butyrophenone and thioxanthene neuroleptics
B.1	T43.5	Poisoning by psychotropic drugs, not elsewhere classified: Other and unspecified antipsychotics and neuroleptics
B.1	T43.6	Poisoning by psychotropic drugs, not elsewhere classified: Psychostimulants with abuse potential
B.1	T43.8	Poisoning by psychotropic drugs, not elsewhere classified: Other psychotropic drugs, not elsewhere classified
B.1	T43.9	Poisoning by psychotropic drugs, not elsewhere classified: Psychotropic drug, unspecified
B.1	T44	Poisoning by drugs primarily affecting the autonomic nervous system:
B.1	T44.0	Poisoning by drugs primarily affecting the autonomic nervous system: Other parasympathomimetics [cholinergics]
B.1	T44.1	Poisoning by drugs primarily affecting the autonomic nervous system: Other parasympathomimetics [cholinergics]
B.1	T44.2	Poisoning by drugs primarily affecting the autonomic nervous system: Ganglionic blocking drugs, not elsewhere classified
B.1	T44.3	Poisoning by drugs primarily affecting the autonomic nervous system:Other parasympatholytics [anticholinergics and antimuscarinics] and spasmolytics, not elsewhere classified
B.1	T44.4	Poisoning by drugs primarily affecting the autonomic nervous system: Predominantly alpha-adrenoreceptor agonists, not elsewhere classified
B.1	T44.5	Poisoning by drugs primarily affecting the autonomic nervous system: Predominantly beta-adrenoreceptor agonists, not elsewhere classified
B.1	T44.6	Poisoning by drugs primarily affecting the autonomic nervous system: Alpha-adrenoreceptor antagonists, not elsewhere classified
B.1	T44.7	Poisoning by drugs primarily affecting the autonomic nervous system: Beta-adrenoreceptor antagonists, not elsewhere classified
B.1	T44.8	Poisoning by drugs primarily affecting the autonomic nervous system: Centrally acting and adrenergic-neuron-blocking agents, not elsewhere classified
B.1	T44.9	Poisoning by drugs primarily affecting the autonomic nervous system: Other and unspecified drugs primarily affecting the autonomic nervous system
B.1	T45	Poisoning by primarily systemic and haematological agents, not elsewhere classified
B.1	T45.0	Poisoning by primarily systemic and haematological agents, not elsewhere classified: Antiallergic and antiemetic drugs
B.1	T45.1	Poisoning by primarily systemic and haematological agents, not elsewhere classified: Antineoplastic and immunosuppressive drugs
B.1	T45.2	Poisoning by primarily systemic and haematological agents, not elsewhere classified: Vitamins, not elsewhere classified
B.1	T45.3	Poisoning by primarily systemic and haematological agents, not elsewhere classified: Enzymes, not elsewhere classified
B.1	T45.4	Poisoning by primarily systemic and haematological agents, not elsewhere classified: Iron and its compounds
B.1	T45.5	Poisoning by primarily systemic and haematological agents, not elsewhere classified: Anticoagulants
B.1	T45.6	Poisoning by primarily systemic and haematological agents, not elsewhere classified: Fibrinolysis-affecting drugs
B.1	T45.7	Poisoning by primarily systemic and haematological agents, not elsewhere classified: Anticoagulant antagonists, vitamin K and other coagulants
B.1	T45.8	Poisoning by primarily systemic and haematological agents, not elsewhere classified: Other primarily systemic and haematological agents
B.1	T45.9	Poisoning by primarily systemic and haematological agents, not elsewhere classified: Primarily systemic and haematological agent, unspecified
B.1	T46	Poisoning by agents primarily affecting the cardiovascular system
B.1	T46.0	Poisoning by agents primarily affecting the cardiovascular system: Cardiac-stimulant glycosides and drugs of similar action
B.1	T46.1	Poisoning by agents primarily affecting the cardiovascular system: Calcium-channel blockers
B.1	T46.2	Poisoning by agents primarily affecting the cardiovascular system: Other antidysrhythmic drugs, not elsewhere classified
B.1	T46.3	Poisoning by agents primarily affecting the cardiovascular system: Coronary vasodilators, not elsewhere classified
B.1	T46.4	Poisoning by agents primarily affecting the cardiovascular system: Angiotensin-converting-enzyme inhibitors
B.1	T46.5	Poisoning by agents primarily affecting the cardiovascular system: Other antihypertensive drugs, not elsewhere classified
B.1	T46.6	Poisoning by agents primarily affecting the cardiovascular system: Antihyperlipidaemic and antiarteriosclerotic drugs

B.1	T46.7	Poisoning by agents primarily affecting the cardiovascular system: Peripheral vasodilators
B.1	T46.8	Poisoning by agents primarily affecting the cardiovascular system: Antivaricose drugs, including sclerosing agents
B.1	T46.9	Poisoning by agents primarily affecting the cardiovascular system: Other and unspecified agents primarily affecting the cardiovascular system
B.1	T47	Poisoning by agents primarily affecting the gastrointestinal system
B.1	T47.0	Poisoning by agents primarily affecting the gastrointestinal system: Histamine H2-receptor antagonists
B.1	T47.1	Poisoning by agents primarily affecting the gastrointestinal system: Other antacids and anti-gastric-secretion drugs
B.1	T47.2	Poisoning by agents primarily affecting the gastrointestinal system: Stimulant laxatives
B.1	T47.3	Poisoning by agents primarily affecting the gastrointestinal system: Saline and osmotic laxatives
B.1	T47.4	Poisoning by agents primarily affecting the gastrointestinal system: Other laxatives
B.1	T47.5	Poisoning by agents primarily affecting the gastrointestinal system: Digestants
B.1	T47.6	Poisoning by agents primarily affecting the gastrointestinal system: Antidiarrhoeal drugs
B.1	T47.7	Poisoning by agents primarily affecting the gastrointestinal system: Emetics
B.1	T47.8	Poisoning by agents primarily affecting the gastrointestinal system: Other agents primarily affecting the gastrointestinal system
B.1	T47.9	Poisoning by agents primarily affecting the gastrointestinal system: Agent primarily affecting the gastrointestinal system, unspecified
B.1	T48	Poisoning by agents primarily acting on smooth and skeletal muscles and the respiratory system
B.1	T48.0	Poisoning by agents primarily acting on smooth and skeletal muscles and the respiratory system: Oxytocic drugs
B.1	T48.1	Poisoning by agents primarily acting on smooth and skeletal muscles and the respiratory system: Skeletal muscle relaxants [neuromuscular blocking agents]
B.1	T48.2	Poisoning by agents primarily acting on smooth and skeletal muscles and the respiratory system: Other and unspecified agents primarily acting on muscle
B.1	T48.3	Poisoning by agents primarily acting on smooth and skeletal muscles and the respiratory system: Antitussive
B.1	T48.4	Poisoning by agents primarily acting on smooth and skeletal muscles and the respiratory system: Expectorants
B.1	T48.5	Poisoning by agents primarily acting on smooth and skeletal muscles and the respiratory system: Anti-common-cold drugs
B.1	T48.6	Poisoning by agents primarily acting on smooth and skeletal muscles and the respiratory system: Antiasthmatics, not elsewhere classified
B.1	T48.7	Poisoning by agents primarily acting on smooth and skeletal muscles and the respiratory system: Other and unspecified agents primarily acting on the respiratory system
B.1	T49	Poisoning by topical agents primarily affecting skin and mucous membrane and by ophthalmological, otorhinolaryngological and dental drugs
B.1	T49.0	Poisoning by topical agents primarily affecting skin and mucous membrane and by ophthalmological, otorhinolaryngological and dental drugs: Local antifungal, anti-infective and anti-inflammatory drugs, not elsewhere classified
B.1	T49.1	Poisoning by topical agents primarily affecting skin and mucous membrane and by ophthalmological, otorhinolaryngological and dental drugs: Antipruritics
B.1	T49.2	Poisoning by topical agents primarily affecting skin and mucous membrane and by ophthalmological, otorhinolaryngological and dental drugs: Local astringents and local detergents
B.1	T49.3	Poisoning by topical agents primarily affecting skin and mucous membrane and by ophthalmological, otorhinolaryngological and dental drugs: Emollients, demulcents and protectants
B.1	T49.4	Poisoning by topical agents primarily affecting skin and mucous membrane and by ophthalmological, otorhinolaryngological and dental drugs: Keratolytics, keratoplastics and other hair treatment drugs and preparations
B.1	T49.5	Poisoning by topical agents primarily affecting skin and mucous membrane and by ophthalmological, otorhinolaryngological and dental drugs: Ophthalmological drugs and preparations
B.1	T49.6	Poisoning by topical agents primarily affecting skin and mucous membrane and by ophthalmological, otorhinolaryngological and dental drugs: Otorhinolaryngological drugs and preparations
B.1	T49.7	Poisoning by topical agents primarily affecting skin and mucous membrane and by ophthalmological, otorhinolaryngological and dental drugs: Dental drugs, topically applied
B.1	T49.8	Poisoning by topical agents primarily affecting skin and mucous membrane and by ophthalmological, otorhinolaryngological and dental drugs: Other topical agents
B.1	T49.9	Poisoning by topical agents primarily affecting skin and mucous membrane and by ophthalmological, otorhinolaryngological and dental drugs: Topical agent, unspecified
B.1	T50	Poisoning by diuretics and other and unspecified drugs, medicaments and biological substances
B.1	T50.0	Poisoning by diuretics and other and unspecified drugs, medicaments and biological substances: Mineralocorticoids and their antagonists
B.1	T50.1	Poisoning by diuretics and other and unspecified drugs, medicaments and biological substances: Loop [high-ceiling] diuretics
B.1	T50.2	Poisoning by diuretics and other and unspecified drugs, medicaments and biological substances: Carbonic-anhydrase inhibitors, benzothiadiazides and other diuretics
B.1	T50.4	Poisoning by diuretics and other and unspecified drugs, medicaments and biological substances: Drugs affecting uric acid metabolism
B.1	T50.6	Poisoning by diuretics and other and unspecified drugs, medicaments and biological substances: Antidotes and chelating agents, not elsewhere classified

B.1	T50.7	Poisoning by diuretics and other and unspecified drugs, medicaments and biological substances: Analeptics and opioid receptor antagonists
B.2	F55	Abuse of non-dependence-producing substances
B.2	F55.0	Abuse of non-dependence-producing substances: Antidepressants
B.2	F55.1	Abuse of non-dependence-producing substances: Laxatives
B.2	F55.2	Abuse of non-dependence-producing substances: Analgesics
B.2	F55.3	Abuse of non-dependence-producing substances: Antacids
B.2	F55.4	Abuse of non-dependence-producing substances: Vitamins
B.2	F55.5	Abuse of non-dependence-producing substances: Steroids and hormones
B.2	F55.6	Abuse of non-dependence-producing substances: Herbal or folk remedies
B.2	F55.8	Abuse of non-dependence-producing substances, other non-dependence-producing substances
B.2	F55.9	Abuse of non-dependence-producing substances: Unspecified
B.2	T50.3	Poisoning by diuretics and other and unspecified drugs, medicaments and biological substances: Electrolytic, caloric and water-balance agents
B.2	T50.5	Poisoning by diuretics and other and unspecified drugs, medicaments and biological substances: Appetite depressants
B.2	T50.8	Poisoning by diuretics and other and unspecified drugs, medicaments and biological substances: Diagnostic agents
B.2	T50.9	Poisoning by diuretics and other and unspecified drugs, medicaments and biological substances: Other and unspecified drugs, medicaments and biological substances
B.2	T96	Sequelae of poisoning by drugs, medicaments and biological substances
C	A04.7	Enterocolitis due to Clostridium difficile
C	D69.0	Allergic purpura
C	D69.2	Other nonthrombocytopenic purpura
C	D69.5	Secondary thrombocytopenia
C	D69.57	Secondary thrombocytopenia, refractory to transfusions
C	D69.58	Secondary thrombocytopenia, not refractory to transfusions
C	D69.59	Secondary thrombocytopenia, unspecified
C	E15	Nondiabetic hypoglycaemic coma
C	H91.0	Ototoxic hearing loss
C	K52.1	Toxic gastroenteritis and colitis
C	K71	Toxic liver disease
C	K71.0	Toxic liver disease with cholestasis
C	K71.1	Toxic liver disease with hepatic necrosis
C	K71.2	Toxic liver disease with acute hepatitis
C	K71.3	Toxic liver disease with chronic persistent hepatitis
C	K71.4	Toxic liver disease with chronic lobular hepatitis
C	K71.5	Toxic liver disease with chronic active hepatitis
C	K71.6	Toxic liver disease with hepatitis, not elsewhere classified
C	K71.7	Toxic liver disease with fibrosis and cirrhosis of liver
C	K71.8	Toxic liver disease with other disorders of liver
C	K71.9	Toxic liver disease, unspecified
C	L51	Erythema multiforme
C	L51.0	Nonbullous erythema multiforme
C	L51.1	Bullous erythema multiforme
C	L51.2	Toxic epidermal necrolysis [Lyell]
C	L51.20	Toxic epidermal necrolysis, Infestation of less than 30 % of the body surface
C	L51.21	Toxic epidermal necrolysis, Infestation of 30 % of the body surface
C	L51.8	Other erythema multiforme
C	L51.9	Erythema multiforme, unspecified
C	L56.2	Photocontact dermatitis

C	N99.0	Postprocedural renal failure
C	O74.2	Cardiac complications of anaesthesia during labour and delivery
C	O74.3	Central nervous system complications of anaesthesia during labour and delivery
C	Y69	Unspecified misadventure during surgical and medical care
D	K25	Gastric ulcer
D	K25.0	Gastric ulcer: Acute with haemorrhage
D	K25.1	Gastric ulcer: Acute with perforation
D	K25.2	Gastric ulcer: Acute with both haemorrhage and perforation
D	K25.3	Gastric ulcer: Acute without haemorrhage or perforation
D	K25.4	Gastric ulcer: Chronic or unspecified with haemorrhage
D	K25.5	Gastric ulcer: Chronic or unspecified with perforation
D	K25.6	Gastric ulcer: Chronic or unspecified with both haemorrhage and perforation
D	K25.7	Gastric ulcer: Chronic without haemorrhage or perforation
D	K25.9	Gastric ulcer: Unspecified as acute or chronic, without haemorrhage or perforation
D	K26	Duodenal ulcer
D	K26.0	Duodenal ulcer: Acute with haemorrhage
D	K26.1	Duodenal ulcer: Acute with perforation
D	K26.2	Duodenal ulcer: Acute with both haemorrhage and perforation
D	K26.3	Duodenal ulcer: Acute without haemorrhage or perforation
D	K26.4	Duodenal ulcer: Chronic or unspecified with haemorrhage
D	K26.5	Duodenal ulcer: Chronic or unspecified with perforation
D	K26.6	Duodenal ulcer: Chronic or unspecified with both haemorrhage and perforation
D	K26.7	Duodenal ulcer: Chronic without haemorrhage or perforation
D	K26.9	Duodenal ulcer: Unspecified as acute or chronic, without haemorrhage or perforation
D	K27	Peptic ulcer, site unspecified
D	K27.0	Peptic ulcer, site unspecified: Acute with haemorrhage
D	K27.1	Peptic ulcer, site unspecified: Acute with perforation
D	K27.2	Peptic ulcer, site unspecified: Acute with both haemorrhage and perforation
D	K27.3	Peptic ulcer, site unspecified: Acute without haemorrhage or perforation
D	K27.4	Peptic ulcer, site unspecified: Chronic or unspecified with haemorrhage
D	K27.5	Peptic ulcer, site unspecified: Chronic or unspecified with perforation
D	K27.6	Peptic ulcer, site unspecified: Chronic or unspecified with both haemorrhage and perforation
D	K27.7	Peptic ulcer, site unspecified: Chronic without haemorrhage or perforation
D	K27.9	Peptic ulcer, site unspecified: Unspecified as acute or chronic, without haemorrhage or perforation
D	K28	Gastrojejunal ulcer
D	K28.0	Gastrojejunal ulcer: Acute with haemorrhage
D	K28.1	Gastrojejunal ulcer: Acute with perforation
D	K28.2	Gastrojejunal ulcer: Acute with both haemorrhage and perforation
D	K28.3	Gastrojejunal ulcer: Acute without haemorrhage or perforation
D	K28.4	Gastrojejunal ulcer: Chronic or unspecified with haemorrhage
D	K28.5	Gastrojejunal ulcer: Chronic or unspecified with perforation
D	K28.6	Gastrojejunal ulcer: Chronic or unspecified with both haemorrhage and perforation
D	K28.7	Gastrojejunal ulcer: Chronic without haemorrhage or perforation
D	K28.9	Gastrojejunal ulcer: Unspecified as acute or chronic, without haemorrhage or perforation
D	K29.0	Gastritis and duodenitis
D	L50.0	Allergic urticaria
D	N17	Acute renal failure

D	N17.0	Acute renal failure with tubular necrosis
D	N17.1	Acute renal failure with acute cortical necrosis
D	N17.2	Acute renal failure with medullary necrosis
D	N17.8	Other acute renal failure
D	N17.9	Acute renal failure, unspecified
E	I26.0	Pulmonary embolism with mention of acute cor pulmonale
E	I61.0	Intracerebral haemorrhage in hemisphere, subcortical
E	I61.1	Intracerebral haemorrhage in hemisphere, cortical
E	I61.2	Intracerebral haemorrhage in hemisphere, unspecified
E	I61.3	Intracerebral haemorrhage in brain stem
E	I61.4	Intracerebral haemorrhage in cerebellum
E	I61.5	Intracerebral haemorrhage, intraventricular
E	I61.6	Intracerebral haemorrhage, multiple localized
E	I61.8	Other intracerebral haemorrhage
E	I61.9	Intracerebral haemorrhage, unspecified
E	J45.0	Predominantly allergic asthma
E	K72.0	Acute and subacute hepatic failure
E	K92.2	Gastrointestinal haemorrhage, unspecified
